# Loading Effect of Sol-Gel Derived Barium Hexaferrite on Magnetic Polymer Composites

**DOI:** 10.3390/nano11030558

**Published:** 2021-02-24

**Authors:** Thanida Charoensuk, Wannisa Thongsamrit, Chesta Ruttanapun, Pongsakorn Jantaratana, Chitnarong Sirisathitkul

**Affiliations:** 1 Thailand Center of Excellence in Physics, Ministry of Higher Education, Science, Research and Innovation, 328 Si Ayutthaya Road, Bangkok 10400, Thailand; cthanida@mail.wu.ac.th; 2 Department of Physics, Faculty of Science, King Mongkut’s Institute of Technology Ladkrabang, Chalongkrung Road, Ladkrabang, Bangkok 10520, Thailand; 61605076@kmitl.ac.th (W.T.); chesta.ru@kmitl.ac.th (C.R.); 3 Center of Excellence in Smart Materials Research and Innovation, King Mongkut’s Institute of Technology Ladkrabang, Chalongkrung Road, Ladkrabang, Bangkok 10520, Thailand; 4 Department of Physics, Faculty of Science, Kasetsart University, 50 Ngam Wong Wan Road, Chatuchak, Bangkok 10900, Thailand; fscipsj@ku.ac.th; 5 Functional Materials and Nanotechnology Center of Excellence, Walailak University, Nakhon Si Thammarat 80160, Thailand; 6 Division of Physics, School of Science, Walailak University, Nakhon Si Thammarat 80160, Thailand

**Keywords:** magnetic polymer composite, sol-gel auto-combustion, acrylonitrile-butadiene-styrene, barium hexaferrite, rare-earth-free magnet

## Abstract

Solution–processing methods were investigated as viable alternatives to produce the polymer-bonded barium hexaferrite (BaM). BaM powders were first synthesized by using the sol-gel auto-combustion method. While the ignition period in two synthesis batches varied, the morphology of hexagonal microplates and nanorods, as well as magnetic properties, were reproduced. To prepare magnetic polymer composites, these BaM powders were then incorporated into the acrylonitrile-butadiene-styrene (ABS) matrix with a weight ratio of 80:20, 70:30, and 60:40 by using the solution casting method. Magnetizations were linearly decreased with a reduction in ferrite loading. Compared to the BaM loose powders and pressed pellet, both remanent and saturation magnetizations were lower and gave rise to comparable values of the squareness. The squareness around 0.5 of BaM samples and their composites revealed the isotropic alignment. Interestingly, the coercivity was significantly increased from 1727–1776 Oe in loose BaM powders to 1874–2052 Oe for the BaM-ABS composites. These composites have potential to be implemented in the additive manufacturing of rare-earth-free magnets.

## 1. Introduction

The developments of polymer-bonded magnets were initially focused on NdFeB [1,2,3,4,5,6] as well as SmCo [7]. Recent attention has been paid to rare-earth-free magnets like manganese alloys [8] and hard ferrites [3,9]. The M-type barium hexaferrite (BaFe_12_O_19_ or BaM) exhibits ferrimagnetism, which has been deployed in low-cost permanent magnets [10]. In addition to hard magnetic properties with large magneto-crystalline anisotropy, high Curie temperature, high saturation magnetization, and coercivity, the BaM has excellent chemical stability, corrosion resistivity, and low cost [10,11,12,13]. These characteristics remarkably fulfill the requirement of rare-earth-free permanent magnets. Several techniques have therefore been investigated to synthesize the high-performance BaM, i.e., hydrothermal synthesis [14], carbon combustion synthesis of oxides [15], reverse microemulsion technique [16], co-precipitation-calcination [17,18], oxalate precursor method [19], and sol-gel based combustion [11,12,13,20,21,22,23,24,25,26,27,28].

In the sol-gel based combustion technique, barium nitrate and iron nitrate were commonly used as precursors with a fuel that also functions as a chelating agent, e.g., citric acid [13,20,21,23,26] and some carbohydrate sugars [12,22]. The conditions of the sol-gel auto-combustion [13,20,21,22,23,25], the citrate-EDTA complexing method [24], and the sol-gel method without water and surfactants [27] were specifically studied to enhance the hard magnetic properties. For the sol-gel auto-combustion process, a stoichiometric amount of metal nitrate and fuel [13], pH effect [21], and amount of Fe^3+^/Ba^2+^ ratio, causing the impurity phase formation [22,23,24], were influential. Magnetic properties could be improved by doping Sr, Sm, Tm, Co, and Cu into crystalline structures during the sol-gel syntheses [11,20,27].

The BaM phase formation, morphology, and magnetic properties are also sensitive to the heat treatment on sol-gel derived products. Widyastuti et al. reported that the α–Fe_2_O_3_ phase dominated after calcining at 750 °C and 850 °C, whereas the BaM was dominant as a result of higher temperature calcination at 950 °C. Accordingly, the highest magnetization of 63.5 emu/g and 3542 Oe were obtained after sintering at 950 °C as the particles grew from the average size around 0.2 to 0.7 μm with increasing sintering temperatures [23]. Wang and Zhang suggested that higher temperature and longer holding times promoted crystal grain growth. By sintering at 1000 °C for 5 h, hexagonal microplates of BaM with a grain size of around 1–2 μm were obtained. The Differential Scanning Calorimetry (DSC) revealed two exothermic peaks at about 380 °C related to the decomposition of remaining esters and 850 °C attributed to the formation of BaM [24]. Mali and Ataie showed that the amorphous dried gel transformed to the crystalline powder after the combustion, and the pure BaM phase was obtained after calcination at 900 °C. Besides, the increase in sintering temperature from 1000 °C to 1100 °C resulted in the agglomeration of spherical particles of 0.4 μm in diameter into the hexagonal microplates with an average size of around 2.5 μm [25].

Additive manufacturing is increasingly important in many technologies because novel products of complex shape can be fabricated with less weight and minimal wastes [1,29,30,31,32]. Its versatilities have been demonstrated in the direct 3D metal printing of permanent magnets of varying shapes and sizes by selective laser melting [33,34], laser beam melting [35], electron beam melting [29,36], and binder jet [37]. Moreover, the fused deposition modeling (FDM) can facilely produce the polymer-bonded magnets with a complex net-shape and functionality, becoming an alternative to the injection molding commonly available for industrial productions [1,22,23,24,25,30,38]. Common 3D-printing is compatible with magnetic polymer composites in the form of filaments [39]. Besides, some 3D-printers have been developed for production with polymer-bonded composites in the form of granules [40]. It follows that the investigation on printable magnetic polymer composites has been intensified. Thermoplastics, such as polylactic acid (PLA) [7], polyamide (PA) [1,2,5], polyethylene (PE) [8], polyphenylene sulfide (PPS) [4], acrylonitrile-butadiene-styrene (ABS) [9], ethylene ethyl acrylate (EEA) [2], and nylon [6], were tested as the polymeric matrix for embedding magnetic powders. Predefined external field [5,41] and big area additive manufacturing [5,42] techniques were also developed to improve the performance of polymer-bonded magnets.

In this research, BaM-ABS composites are fabricated for further uses in the additive manufacturing of rare-earth-free magnets. Following requirements in industrial productions, the reproducibility of magnetic properties of BaM from the sol-gel auto-combustion is firstly investigated. The solution-processing method is also used to incorporate sol-gel derived BaM powders in ABS granules. While the largest ferrite loading straightforwardly leads to the largest magnetization, the homogeneity and mechanical properties need to be considered [9,30]. The incorporation of magnetic powders affects the viscosity of the polymeric matrix, which limits the working in additive manufacturing and the loadings of 45–65% are typically used in the case of magnetic thermoplastic filaments [29]. The variations of BaM from 60% to 80% by weight are selected for this research because, from the preliminary test, these loadings led to printable magnetic polymer composites.

## 2. Materials and Methods

### 2.1. Synthesis and Characterization of BaM Powders

**Figure 1:**
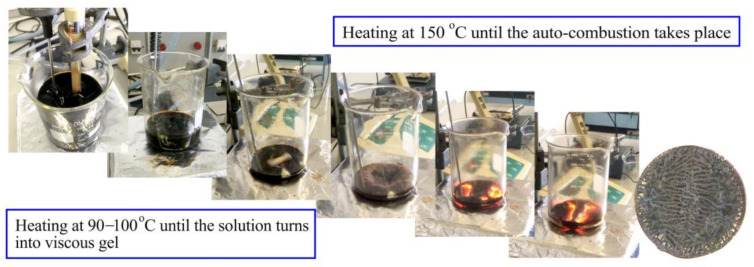
Stepwise photographs of sol-gel auto-combustion synthesis of BaM nanoparticles.

In the sol-gel auto-combustion synthesis of BaM, the molar composition of iron(III) nitrate nonahydrate (Fe(NO_3_)_3_·9H_2_O) (Sigma-Aldrich, Singapore: ACS reagent, 98.0–101.0%), barium nitrate (Ba(NO_3_)_2_) (HIMEDIA, India: ACS 99.00–102.00%) and citric acid (C_3_H_5_O(COOH)_3_ was 12:1:0.75. Following the suitable condition for forming a single-phase BaM in the previous reports [17,19,23,24], the mole ratio of Ba to Fe in the precursors was selected as 12:1. The citric acid acted as a chelating agent in the bonding of anions (citrate^(3−)^ and citrate^(2−)^) to the Fe^3+^ and Ba^2+^ cations. The starting reagents were successively dissolved in deionized (DI) water with a mass (g) per volume (mL) ratio of 1:6. Under constant magnetic stirring, the aqueous acidic solution was obtained. The pH was adjusted to 7 by adding ammonium hydroxide (27% NH_4_OH). The solution was then heated and continuously stirred at 90–100 °C for around 3 h to evaporate the solvents. The viscous brown gel was formed and eventually dried. Afterwards, the temperature was promptly increased to 150 °C. As a result, the dried gel swelled up until reaching ignition. The auto-combustion took place and propagated throughout the gel within a minute. The obtained brown powders were ground and then heated at 450 °C for 2 h to remove residual carbon compounds [13,20]. The subsequent calcination at 1050 °C for 3 h finally allowed for the formation of BaM [10,20]. The changes from the solution to the final solid product are shown in Figure 1.

To verify the reproducibility of the sol-gel auto-combustion, two batches of BaM, referred to as BaM_ex1 and BaM_ex2, were prepared by using the same procedure. It was noted that the combustion step might have occurred before the gel was completely dried. As a result of the wet gel ignition, the ignition period of 1 min for the BaM_ex2 sample was much longer than the 20 s observed in the BaM_ex1 sample. Morphology and elemental compositions of both samples were, respectively, examined by the field-emission scanning electron microscope (FESEM: Zeiss Merlin Compact, Carl Zeiss Microscopy GmbH, Germany) and the energy-dispersive X-ray spectroscopy (EDS: Oxford Aztec, Oxford Instruments, UK). The ferrite phases were identified by using a single crystal X-ray diffractometer (XRD, Rigaku SuperNova diffractometer with a HyPix 3000 detector, Rigaku Corporation, Poland) using Cuα radiation (λ = 1.54184 Å). Data reduction, scaling and absorption corrections were performed using CrysAlisPro software in the micro powder mode. A vibrating sample magnetometer (VSM: in-house developed and calibrated with Lakeshore 730,908 using a Ni sphere of 3 mm in diameter) was used to measure the magnetization (*M*) as a function of the external magnetic field (*H*) between −17,500 and 17,500 Oe.

### 2.2. Synthesis and Characterization of BaM-ABS Composites

To prepare the magnetic polymer composites for further uses in additive manufacturing of rare-earth-free magnets, the sol-gel derived BaM powders were incorporated into the ABS matrix via the solution casting method. Firstly, the ABS pellets (GA800, Polimaxx, IRPC Public Company Limited, Thailand) were completely dissolved in acetone under constant stirring with a magnetic bar for 2 h at room temperature. Then, the BaM powders were added to the solution, and the stirring continued for 1 h to thoroughly disperse the BaM in the ABS. 20 mL acetone was required for 2.5 g of BaM and ABS in total. After that, the residual acetone was eliminated by evaporation. The product was dried at room temperature for 24 h, resulting in the BaM in the ABS matrix. The weight ratios of BaM:ABS varied as 60:40, 70: 30, 80:20, and magnetic properties of these composites, respectively referred to as BaM60-ABS40, BaM70-ABS30, and BaM80-ABS20, were measured by the VSM. In addition, a gram of BaM powder was pressed under a uniaxial pressure at 60 kg/cm^3^ for 5 min with an automatic hydraulic machine to compare the magnetic properties of this BaM pellet with those of the BaM-ABS composites. From hysteresis loops, the coercivity (*H_c_*) and the remanent magnetization (*M_r_*) were, respectively, determined from the *x*-intercept and the *y*-intercept.

## 3. Results and Discussion

### 3.1. Characterization of BaM Powders

**Figure 2:**
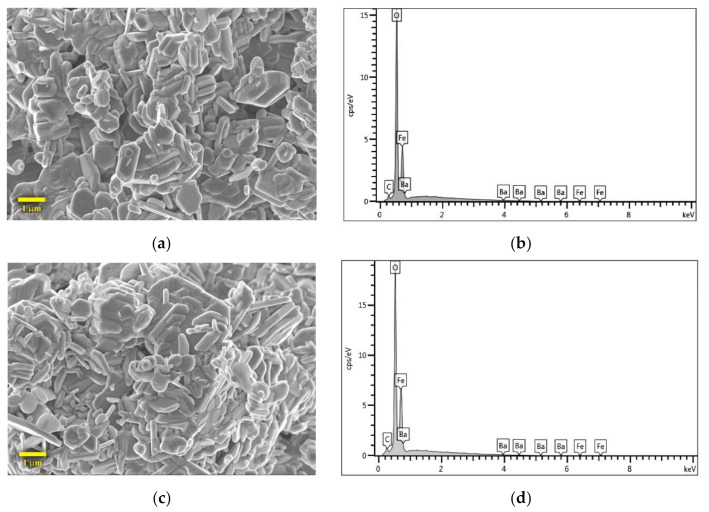
Morphology and elemental composition of sol-gel derived products after the calcination; (**a**) FESEM image and (**b**) EDS spectra of BaM_ex1; (**c**) FESEM image and (**d**) EDS spectra BaM_ex2.

The sol-gel derived products after calcining at 1050 °C for 3 h revealed the morphology, as shown in Figure 2a,c. Both BaM_ex1 and BaM_ex2 samples are similarly composed of hexagonal plates and nanorods. The size of hexagonal plates ranges from less than 0.3 μm to over 1 μm. Compared to the previous reports on BaM, Mali and Ataie obtained hexagonal plates with an average size of about 2.5 μm after the calcination at 1100 °C for 1 h [25] and Wang and Zhang observed the hexagonal microplate with a grain size of 1–2 μm from the powder received heat treatment at 1000 °C for 5 h [24]. The EDS spectra in Figure 2b,d reveal peaks corresponding to Fe, Ba, O, and C elements. Since an artificial carbon peak is normally visible on EDS spectra, these spectra are consistent with the composition of BaM. However, measurements at some other points do not yield a clear Ba peak, indicating inhomogeneity. The phase composition is therefore not conclusive from the EDS results and other minor phases cannot be ruled out. The calcination temperature at 1050 °C tends to promote the pure phase BaFe_12_O_19_ formation [28], and the appropriate high temperature and holding time for calcination significantly affect the crystalline structure of BaM [24,25]. While the DSC, differential thermal analysis (DTA) and thermogravimetric analysis (TGA) spectra revealed the BaM phase formation at 840–860 °C [24,25,26], the calcinations at higher temperatures were reported to promote the hexagonal structures as well as the crystalline size of BaM [17,19,25].

**Figure 3:**
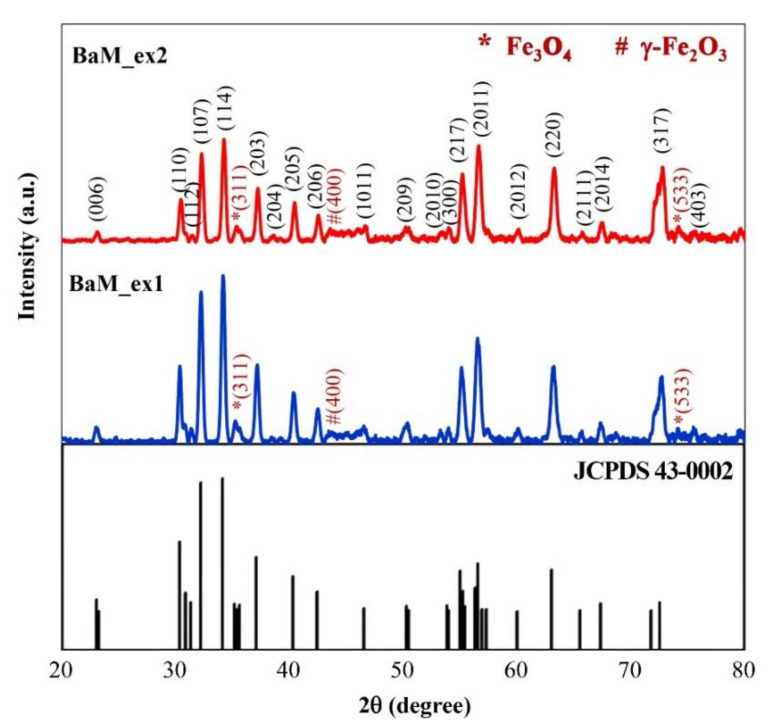
XRD patterns of sol-gel derived products after calcination.

The phase implied by the EDS results is confirmed by XRD. In Figure 3, the spectra from both BaM_ex1 and BaM_ex2 samples exhibit characteristic peaks of the M-type BaFe_12_O_19_ (JCPDS 43-0002). The diffraction peaks at 23.0°, 30.3°, 31.3°, 32.2°, 34.1°, 37.1°, 38.5°, 40.3°, 42.4°, 46.6°, 50.3°, 53.3°, 53.8°, 55.1°, 56.6°, 60.0°, 63.2°, 65.6°, 67.3°, 72.6°, and 75.5° correspond to the crystallographic planes of (006), (110), (112), (107), (114), (203), (204), (205), (206), (1011), (209), (2010), (300), (217), (2011), (2012), (220), (2111), (2014), (317), and (403), respectively. In addition to BaFe_12_O_19_, the minor phase of magnetite (Fe_3_O_4_) are indexed at 35.6°(311) and 74.5°(533) (JCPDS 75-0033) and a low-intensity diffraction peak of maghemite (γ-Fe_2_O_3_) phase is also detected at 43.3°(400) (JCPDS 39-1346).

**Figure 4:**
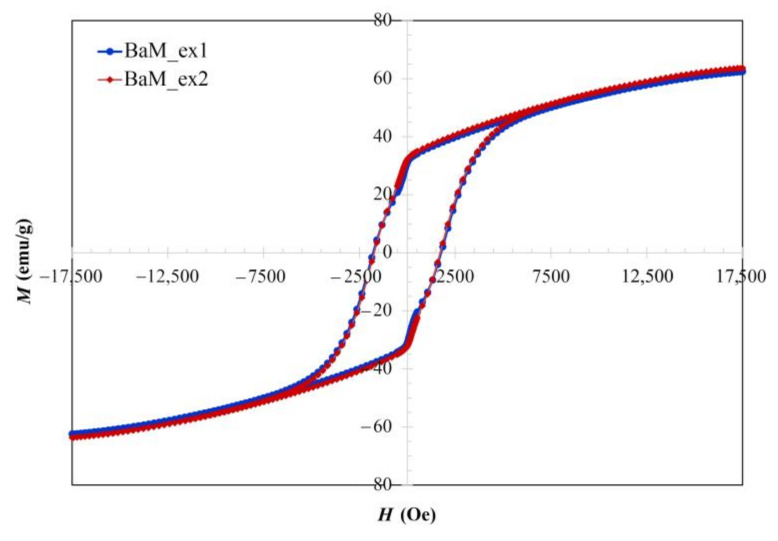
*M-H* curves of BaFe_12_O_19_ powders.

Figure 4 shows similar hysteresis loops from BaM_ex1 and BaM_ex2 samples indicating the reproducibility required for productions of hard magnetic BaM. The solution-processing methods can be scaled up and some ferrite syntheses have been developed at lower temperatures for commercial productions [43]. The maximum magnetizations reach 62.4 and 63.6 emu/g in the applied external magnetic field of 17,500 Oe. The remanent magnetizations are 31.1 and 32.2 emu/g, as listed in Table 1. These magnetizations, as well as the coercivity of about 1750 Oe in Table 1, are functional characteristics of hard magnetic materials [30]. Magnetic properties can be further improved by the varying condition of heat treatments that influences phase purification, crystalline size, and morphology [17,19,25,26].

### 3.2. Magnetic Properties of BaM-ABS Composites

**Figure 5:**
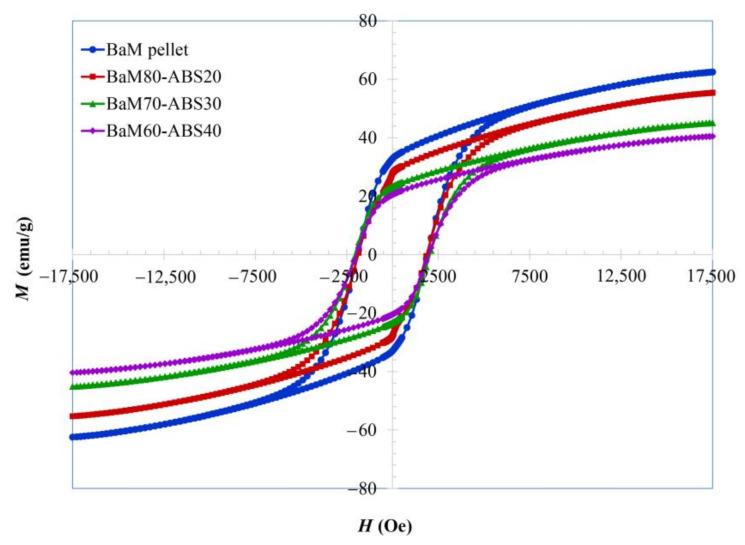
Hysteresis loops of BaM-ABS composites with varying ferrite loading of 60%, 70%, and 80%, as well as a pressed BaM pellet.

Hysteresis loops of BaM-ABS composites are compared to the pressed BaM pellet in Figure 5 and the corresponding magnetic parameters are listed in Table 1. Magnetizations of pressed BaM pellets are almost identical to the value measured in the form of the BaM powder with the remanent magnetization around 32 emu/g. As the magnetization of magnetic polymer composites is only attributed to magnetic filler, the reduction in magnetizations is consistent with the replacement of magnetic materials by the polymeric matrix. Nevertheless, the highest magnetization obtained from the BaM:ABS ratio of 80:20 is as high as 87% of the values in BaM loose powders and pressed pellet.

Unlike magnetizations, the coercivity of the pressed BaM pellet is higher than that of the powder form. The powder compaction induced the stress that influenced the dislocations and associated the lattice distortion, enhancing the coercivity [44,45,46]. Likewise, the polymer-bonded BaM magnets have higher coercivity because of the induced stress [29]. These results are consistent with the reported by El-Sayed et al. on a marked increase in coercivity of the BaM composite in polystyrene [47]. The coercivity differs slightly with the ferrite loading and the BaM:ABS ratio of 70:30 results in the highest coercivity of about 2050 Oe. It follows that the maximum energy product compared in Table 1 is at the highest in the case of this ratio.

As all hysteresis loops in Figure 5 do not reach the saturation in the maximum field supplied, the law of approach to saturation is then used to estimate the saturation magnetizations. With the contribution of magneto-crystalline anisotropy, the change in magnetization at high magnetic fields near the saturation of hard ferrites follows Equation (1) [48,49,50,51,52,53,54]:

(1) $$M\;=\;M_{s}\;\left[1-\frac{B}{H^{2}}\right]$$

where *B* is a constant. Rearranging the terms leads to a linear form in Equation (2).

(2) $$M\;=\;\left(-M_{s}B\right)\cdot \frac{1}{H^{2}}\;+M_{s}\;$$

**Figure 6:**
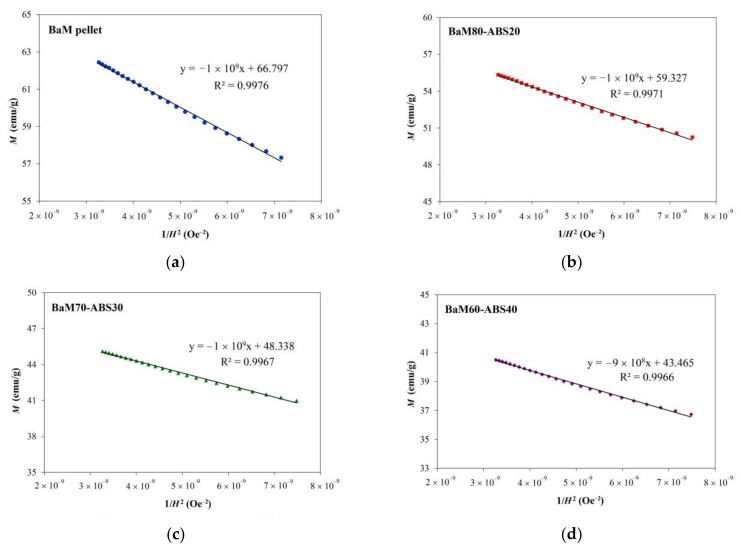
*M* against 1/*H*^2^ plots of (**a**) BaM pellet as well as BaM-ABS composites with varying ferrite loading of (**b**) 80%; (**c**) 70%, and (**d**) 60%.

The plots between *M* and 1/*H*^2^ in a regime approaching 17,500 Oe, the maximum applied field, are shown in Figure 6. All graphs can be linearly fitted, consistent with Equation (2). The saturation magnetization (*M_s_*) is then approximated from the *y*-intercept as listed in Table 2. The slope equals the B value, which is related to the effective anisotropy constant (*K_eff_*) for uniaxial magnetic hexagonal crystals as in Equation (3) [48,49,50,51,52,53,54].

(3) Keff = Ms  15B4 12

The anisotropy field (*H_a_*) can then be calculated by using the *K_eff_* value from Equation (4) [48,49,50,51,52,53,54].

(4) $$H_{a}\;=\;\frac{2K_{eff}}{M_{s}}$$

In Table 2, the saturation magnetizations from the linear fitting are comparable to the literature [20,26,27,55,56,57]. From these experiments, the saturation magnetization from undoped BaM ranges from 49–70 emu/g. Remarkably high magnetizations of 88 and 89 emu/g in BaM were, respectively, obtained from the solid state reaction [58] and ball milling [59]. The *K_eff_*, also listed in Table 2, is significantly reduced with the decreased in ferrite loading. However, the reduction in *M_s_* results in the increase in H_a_ with the decreasing loading from 100% (i.e., a pressed pellet) to 70%. However, the squareness computed from the ratio *M_r_/M_s_* is rather insensitive to the ferrite loading. The value around 0.5 of both BaM and BaM-ABS composites corresponds to the isotropic BaM, commonly found in randomly oriented bulk ferrite magnets [60]. The anisotropic BaM can be produced by heat treatment in the magnetic field. The magnetically oriented grains lead to the squareness close to one along the *c*-axis [10].

**Figure 7:**
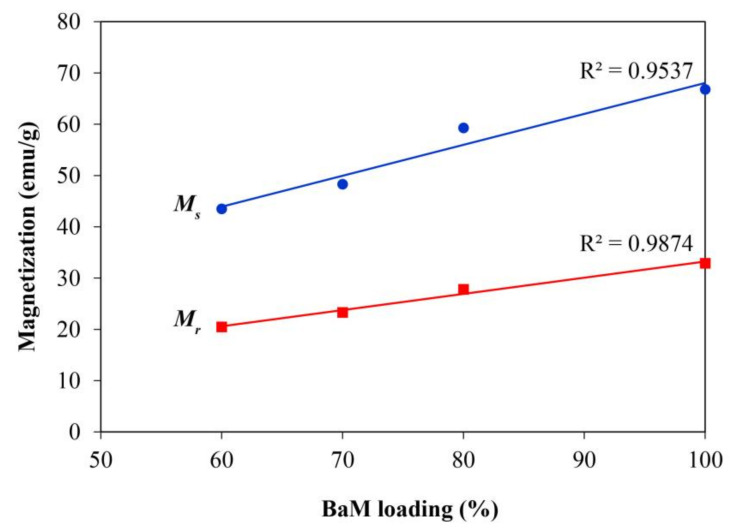
Remanent magnetization (*M_r_*) and saturation magnetization (*M_s_*) as a function of ferrite loading in BaM-ABS composites. The loading of 100% corresponds to the BaM pellet.

In Figure 7, both remanent and saturation magnetizations exhibit linear decreases with the decreasing BaM loading in the composites. Such variations follow the rule of mixture, which was previously applicable to the magnetization of composites with ferrite fillers in either plastic [61] or rubber matrix [62]. In cobalt ferrite-polypropylene composites, the loading of 5–45 wt.% gave rise to a graph fitting passing through the origin [61]. However, the projections of trend lines in Figure 7 predict non-zero remanent and saturation magnetizations of, respectively, 1.66 and 7.73 emu/g at the zero magnetic loading. In the case of remanent magnetization, the R^2^ from the linear fitting is higher and the discrepancy from the line projection is smaller because the values are directly obtained of the *y*-intercept of hysteresis loops. The uncertainty is increased for the saturation magnetization calculated from the law of approach to saturation.

## 4. Conclusions

The M-type BaM were successfully synthesized by the sol-gel auto-combustion. By heating and stirring at 90–100 °C for approximately 3 h, the solution of nitrate nonahydrate, barium nitrate and citric acid transformed into viscous and eventually dried gel. By increasing the temperature to 150 °C, auto-combustion arose and was then terminated within a minute. Despite the variation in the ignition period, magnetic hysteresis loops of BaM from two synthesis batches are almost identical. The results indicated the potential of reproducibility in the sol-gel auto-combustion synthesis for commercial productions. To fabricate magnetic polymer composites, the sol-gel derived BaM was dispersed in the ABS matrix via the solution casting method. The weight ratio of BaM and ABS was varied as 80:20, 70:30, and 60:40 to examine the optimum ferrite filler loading. The excessive loading of BaM may severely deteriorate the mechanic properties desirable in the additive manufacturing, but the loading up to 80% in this research still gave rise to printable composites. All the composites exhibited significant increases in coercivity, while the magnetization values linearly decreased with the reduction in ferrite loading. The highest coercivity of about 2050 Oe was obtained in the BaM-ABS composite with a weight ratio of 70:30. The squareness of every composite was approximately 0.5, indicating the randomly oriented BaM in the ABS matrix.

## Figures and Tables

**Table 1:** Coercivity (*H_c_*), remanent magnetization (*M_r_*), and maximum energy product ((*BH*)_max_) of BaM powders, pressed BaM pellet, and BaM-ABS composites.

Samples	Compositions		Magnetic Properties from Hysteresis Loops		
	BaM (wt%)	ABS (wt%)	*M_r_* (emu/g)	*H_c_* (Oe)	(*BH*)_max_ (MGOe)
BaM_ex1	100	0	31.1	1777	1.30
BaM_ex2	100	0	32.2	1727	1.31
BaM pellet	100	0	32.9	1909	1.39
BaM80-ABS20	80	20	27.8	1874	1.32
BaM70-ABS30	70	30	23.3	2052	1.98
BaM60-ABS40	60	40	20.5	1983	1.31

**Table 2:** Magnetic parameters of pressed BaM pellet and BaM-ABS composites from the linear fitting according to the law of approach to saturation.

Samples	Magnetic Parameters of Linear Fitting			
	*M_s_* (emu/g)	*M* *_r_/M_s_*	*K_eff_* × 10^3^ (emu⋅Oe/g)	*H_a_* (kOe)
BaM pellet	66.8	0.49	500.50	14.98
BaM80-ABS20	59.3	0.47	471.57	15.90
BaM70-ABS30	48.3	0.48	425.59	17.62
BaM60-ABS40	43.5	0.47	383.16	17.62

## Data Availability

The data presented in this study are available on request from the corresponding author.

## References

[1] Huber C., Abert C., Bruckner F., Groenefeld M., Muthsam O., Schuschnigg S., Sirak K., Thanhoffer R., Teliban I., Vogler C. (2016). 3D print of polymer bonded rare-earth magnets, and 3D magnetic field scanning with an end-user 3D printer. Appl. Phys. Lett..

[2] Von Petersdorff-Campen K., Hauswirth Y., Carpenter J., Hagmann A., Boës S., Daners M.S., Penner D., Meboldt M. (2018). 3D printing of functional assemblies with integrated polymer-bonded magnets demonstrated with a prototype of a rotary blood pump. Appl. Sci..

[3] Palmero E.M., Casaleiz D., Jiménez N.A., Rial J., de Vicente J., Nieto A., Altimira R., Bollero A. (2019). Magnetic-polymer composites for bonding and 3D printing of permanent magnets. IEEE Trans. Magn..

[4] Paranthaman M.P., Yildirim V., Lamichhane T.N., Begley B.A., Post B.K., Hassen A.A., Sales B.C., Gandha K., Nlebedim I.C. (2020). Additive manufacturing of isotropic NdFeB PPS bonded permanent magnets. Materials.

[5] Huber C., Abert C., Bruckner F., Groenefeld M., Schuschnigg S., Teliban I., Vogler C., Wautischer G., Windl R., Suess D. (2017). Rare-earth magnets with a variable magnetic compound fraction for a predefined stray field. Sci. Rep..

[6] Li L., Jones K., Sales B., Pries J.L., Nlebedim I.C., Jin K., Bei H., Post B.K., Kesler M.S., Rios O. (2018). Fabrication of highly dense isotropic Nd-Fe-B nylon bonded magnets via extrusion-based additive manufacturing. Addit. Manuf..

[7] Khazdozian H.A., Manzano J.S., Gandha K., Slowing I.I., Nlebedim I.C. (2018). Recycled Sm-Co bonded magnet filaments for 3D printing of magnets. AIP Adv..

[8] Palmero E.M., Rial J., de Vicente J., Camarero J., Skårman B., Vidarsson H., Larsson P.-O., Bollero A. (2018). Development of permanent magnet MnAlC/polymer composites and flexible filament for bonding and 3D-printing technologies. Sci. Technol. Adv. Mater..

[9] Hanemann T., Syperek D., Nötzel D. (2020). 3D printing of ABS barium ferrite composites. Materials.

[10] Pullar R.C. (2012). Hexagonal ferrites: A review of the synthesis, properties and applications of hexaferrite ceramics. Prog. Mater. Sci..

[11] Slimani Y., Almessiere M.A., Güner S., Kurtan U., Baykal A. (2020). Impacts of sol-gel auto-combustion and ultrasonication approaches on structural, magnetic, and optical properties of Sm-Tm Co-substituted Sr_0.5_Ba_0.5_Fe_12_O_19_ nanohexaferrites: Comparative study. Nanomaterials.

[12] Kanagesan S., Jesurani S., Velmurugan R., Kumar C. (2010). Synthesis of barium hexaferrite (BaFe_12_O_19_) using D-fructose as a fuel. J. Manuf. Eng..

[13] Bahadur D., Rajakumar S., Kumar A. (2006). Influence of fuel ratios on auto combustion synthesis of barium ferrite nano particles. J. Chem. Sci..

[14] Chen M., Fan R., Liu G., Wang X., Sun K. (2015). Magnetic properties of barium ferrite prepared by hydrothermal synthesis. Key Eng. Mater..

[15] Martirosyan K.S., Galstyan E., Hossain S.M., Wang Y.-J., Litvinov D. (2011). Barium hexaferrite nanoparticles: Synthesis and magnetic properties. Mater. Sci. Eng..

[16] Xu P., Han X., Wang M. (2007). Synthesis and magnetic properties of BaFe_12_O_19_ hexaferrite nanoparticles by a reverse microemulsion technique. J. Phys. Chem..

[17] Radwan M., Rashad M.M., Hessien M.M. (2007). Synthesis and characterization of barium hexaferrite nanoparticles. J. Mater. Process. Technol..

[18] Ebrahimi Z., Hedayati K., Ghanbari D. (2017). Preparation of hard magnetic BaFe_12_O_19_-TiO_2_ nanocomposites: Applicable for photo-degradation of toxic pollutants. J. Mater. Sci. Mater. Electron..

[19] Mohsen Q. (2010). Factors affecting the synthesis and formation of single-phase barium hexaferrite by a technique of oxalate precursor. Am. J. Appl. Sci..

[20] Asiri S., Güner S., Demir A., Yildiz A., Manikandan A., Baykal A. (2018). Synthesis and magnetic characterization of Cu substituted barium hexaferrites. J. Inorg. Organomet. Polym. Mater..

[21] Xu G., Ma H., Zhong M., Zhou J., Yue Y., He Z. (2006). Influence of pH on characteristics of BaFe_12_O_19_ powder prepared by sol-gel auto-combustion. J. Magn. Magn. Mater..

[22] Mandizadeh S., Soofivand F., Salavati-Niasari M. (2015). Sol-gel auto combustion synthesis of BaFe_12_O_19_ nanoceramics by using carbohydrate sugars as a novel reducing agent. Adv. Powder Technol..

[23] Widyastuti W., Felly Y.F.F., Rochman R., Purwaningsih H. (2011). Effect of Fe^3+^/Ba^2+^ mole ratio and sintering temperatures on the microstructure and magnetic properties of nanoparticle barium hexaferrite (BaM) produced by sol-gel auto combustion. J. Tek. Ind..

[24] Wang L., Zhang Q. (2009). Effect of Fe^3+^/Ba^2+^ mole ratio on the phase formation and microwave properties of BaFe_12_O_19_ prepared by citrate-EDTA complexing method. J. Alloys Compd..

[25] Mali A., Ataie A. (2005). Structural characterization of nano-crystalline BaFe_12_O_19_ powers synthesized by sol-gel combustion route. Scr. Mater..

[26] Shao L.-H., Shen S.-Y., Zheng H., Zheng P., Wu Q., Zheng L. (2018). Effect of powder grain size on microstructure and magnetic properties of hexagonal barium ferrite ceramic. J. Electron. Mater..

[27] Asghar G., Asri S., Khusro S.N., Tariq G.H., Awan M.S., Irshad M., Safeen A., Iqbal Y., Shah W.H., Anis-Ur-Rehman M. (2020). Enhanced magnetic properties of barium hexaferrite. J. Electron. Mater..

[28] Shalini M.G., Subha A., Sahu B., Sahoo S.C. (2019). Phase evolution and temperature dependent magnetic properties of nanocrystalline barium hexaferrite. J. Mater. Sci. Mater. Electron..

[29] Popov V., Koptyug A., Radulov I., Maccari F., Muller G. (2018). Prospects of additive manufacturing of rare-earth and non-rare-earth permanent magnets. Procedia Manuf..

[30] Périgo E.A., Jacimovic J., Ferré F.G., Scherf L.M. (2019). Additive manufacturing of magnetic materials. Addit. Manuf..

[31] Brito-Pereira R., Ribeiro C., Peřinka N., Lanceros-Mendez S., Martins P. (2020). Reconfigurable 3D-printable magnets with improved maximum energy product. J. Mater. Chem..

[32] Cicala G., Ognibene G., Portuesi S., Blanco I., Rapisarda M., Pergolizzi E., Recca G. (2018). Comparison of Ultem 9085 used in fused deposition modelling (FDM) with polytherimide blends. Materials.

[33] Jaćimović J., Binda F., Herrmann L.G., Greuter F., Genta J., Calvo M., Tomše T., Simon R.A. (2017). Net shape 3D printed NdFeB permanent magnet. Adv. Eng. Mater..

[34] Jacimmovic J., Christen T., Dénervaud E. (2020). Self-organized giant magnetic structure via additive manufacturing in NdFeB permanent magnets. Addit. Manuf..

[35] Urban N., Meyer A., Keller V., Franke J. (2018). Contribution of additive manufacturing of rare earth material to the increase in performance and resource efficiency of permanent magnets. Appl. Mech. Mater..

[36] Radulov I.A., Popov V.V., Koptyug A., Maccari F., Kovalevsky A., Essel S., Gassmann J., Skokov K.P., Bamberger M. (2019). Production of net-shape Mn-Al permanent magnets by electron beam melting. Addit. Manuf..

[37] Paranthaman M.P., Shafer C.S., Elliott A.M., Siddel D.H., Mcguire M.A., Springfield R.M., Martin J., Fredette R., Ormerod J. (2016). Binder jetting: A novel NdFeB bonded magnet fabrication process. Jom.

[38] Urban N., Kühl A., Glauche M., Franke J. Additive manufacturing of neodymium-iron-boron permanent magnets. Proceedings of the 2018 8th International Electric Drives Production Conference (EDPC).

[39] Blanco I. (2020). The use of composite materials in 3D printing. J. Compos. Sci..

[40] Spath S., Seitz H. (2014). Influence of grain size and grain-size distribution on workability of granules with 3D printing. Int. J. Adv. Manuf. Technol..

[41] Huber C., Abert C., Bruckner F., Pfaff C., Kriwet J., Groenefeld M., Teliban I., Vogler C., Suess D. (2017). Topology optimized and 3D printed polymer-bonded permanent magnets for a predefined external field. J. Appl. Phys..

[42] Li L., Tirado A., Nlebedim I.C., Rios O., Post B., Kunc V., Lowden R.R., Lara-Curzio E., Fredette R., Ormerod J. (2016). Big area additive manufacturing of high performance bonded NdFeB magnets. Sci. Rep..

[43] Diodati S., Walton R.I., Mascotto S., Gross S. (2020). Low-temperature wet chemistry synthetic approaches towards ferrites. Inorg. Chem. Front..

[44] Palmero E.M., Casaleiz D., de Vicente J., Skårman B., Vidarsson H., Larsson P.-O., Bollero A. (2020). Effect of particle size distribution on obtaining novel MnAlC-based permanent magnet composites and flexible filaments for 3D-printing. Addit. Manuf..

[45] Bittner F., Freudenberger J., Schultz L., Woodcock T.G. (2017). The impact of dislocations on coercivity in L1_0_-MnAl. J. Alloys Compd..

[46] Lindquist A.K., Feinberg J.M., Harrison R.J., Loudon J.C., Newell A.J. (2019). The effects of dislocations on crystallographic twins and domain wall motion in magnetite at the Verwey transition. Earth Planets Space.

[47] El-Sayed A.H., Hemeda O.M., Tawfik A., Hamad M.A. (2015). Remarkable magnetic enhancement of type-M hexaferrite of barium in polystyrene polymer. AIP Adv..

[48] Bate G. (1991). Magnetic recording materials since 1975. J. Magn. Magn. Mater..

[49] Awadallah A., Mahmood S.H., Maswadeh Y., Bsoul I., Aloqaily A. (2015). Structural and magnetic properties of vanadium doped M-type barium haxaferrite (BaFe_12-x_V_x_O_19_). IOP Conf. Ser. Mater. Sci. Eng..

[50] Auwal I.A., Güngüneş H., Güner S., Shirsath S.E., Sertkol M., Baykal A. (2016). Structural, magneto-optical properties and cation distribution of SrBi_x_La_x_Y_x_Fe_12-3x_O_19_ (0.0 ≤ x ≤ 0.33) hexaferrites. Mater. Res. Bull..

[51] Topkaya R. (2017). Effect of Zn substitution on temperature dependent magnetic properties of BaFe_12_O_19_ hexaferrites. J. Alloys Compd..

[52] Mohammad A.M. (2020). Synthesis and study the structural and magnetic properties of cobalt substituted strontium hexaferrite. Int. J. Nanoelectron. Mater..

[53] Bate G. (1980). Chapter 7 Recording materials. Handb. Ferromagn. Mater..

[54] Cullity B.D., Graham C.D. (2009). Introduction to Magnetic Materials.

[55] Makled M.H., Sheha E. (2019). An attempt to utilize hard magnetic BaFe_12_O_19_ phase as a cathode for magnesium batteries. J. Electron. Mater..

[56] Behera P., Ravi S. (2019). Influence of Ti-substitution on structural, magnetic and dielectric properties of M-type barium hexaferrite. J. Electron. Mater..

[57] Faisal M., Saeed A., Larik F.A., Ghumro S.A., Rasheed S., Channar P.A. (2018). WOWS sol–gel based synthesis and structural, morphological, electrical and magnetic characterization of Co-Sm doped M-type barium hexaferrite materials. J. Electron. Mater..

[58] Rafiq M.A., Waqar M., Muhammad Q.K., Waleed M., Saleem M., Anwar M.S. (2018). Conduction mechanism and magnetic behavior of Cu doped barium hexaferrite ceramics. J. Mater. Sci. Mater. Electron..

[59] Winatapura D.S., Deswita D., Fisli A., Adi W.A. (2019). Mechanosynthesis, crystal structure, magnetic and absorption properties of Al substituted BaFe_12_O_19_. Jur. Tek..

[60] Kitakami O., Goto K., Sakurai T. (1988). A study of the magnetic domains of isolated fine particles of Ba ferrite. Jpn. J. Appl. Phys..

[61] Hunyek A., Sirisathitkul C. (2011). Electromagnetic and dynamic mechanical properties of extruded cobalt ferrite-polypropylene composites. Polym. Plast. Technol. Eng..

[62] Mohammed E.M., Malini K.A., Joy P.A., Kulkarni S.D., Date S.K., Kurian P., Anantharaman M.R. (2002). Processability, hardness, and magnetic properties of rubber ferrite composites containing manganese zinc ferrites. Plast. Rubber Compos. Macromol. Eng..

